# An Improvement in Flask Presses Used within the Vulcanizer

**Published:** 1891-07

**Authors:** William H. Trueman

**Affiliations:** Philadelphia


### AN IMPROVEMENT IN FLASK PRESSES USED WITHIN THE VULCANIZER.

BY WILLIAM H. TRUEMAN, PHILADELPHIA.

THERE are on the market several flask presses designed for use in the process of packing into the flask vulcanizable gums that, by spring pressure, gradually, as the prepared gum becomes plastic by the heat applied, closes the flask while it is under steam pressure in the vulcanizer. The practical advantage of so doing is generally recognized. The fault I have found with all such presses so far brought to my notice has been that there is not sufficient run to the screw by which the spring is compressed, the flask must be nearly closed before it can be placed in the press; and also that they require special flasks. Both of these objections I have overcome by the following simple and inexpensive device. The presses are made to hold as many flasks at one time as the vulcanizers they are designed for will contain; there is supplied with them for use, when less than the full complement of flasks are to be packed and vulcanized, a metal block, intended to take the place of the flasks not used. This block I have replaced with one a little more than half its height, having fitted to it a pointed screw about a half inch in diameter projecting centrally from its upper surface, and working in a hole drilled and tapped entirely through it. By this means the height of the block can be adjusted to accommodate the flask used, or to allow the flask to enter the press without undue pressure. The pointed end of the screw fits into a countersink usually found on the lower end of the screw of the press, or, where this is absent, one made to receive it. This renders unnecessary the revolving washer there found, and which may be removed, giving that much more run to the screw and increasing its usefulness. The press thus improved will hold most of the flasks now on the market. The device can be applied equally well to all flask presses intended for use within the closed vulcanizer that I have seen. I therefore deem it unnecessary to name them. I would, however, suggest to the makers—and this applies to all—that the wire upon which the lower plate rests and rocks is entirely too small and weak for the service required. It should be of tough steel (not tool steel), and twice the diameter. The hole in the frame through which it passes should be sufficiently far from the end, or so strengthened as to render tearing out impossible. It is better, if either are to give way, that it should be the wire, as it is more readily replaced; it is much better, however, to have the apparatus amply strong for practical use. The pressure required to close the flask as it should be closed, even when carefully applied under favorable conditions, is very much greater than is generally supposed, and these we cannot always command.

I do not consider it wise to vulcanize, trusting to the press closing the flask, but always open the vulcanizer and inspect the flask to see that it is properly closed, and either increase the pressure, or remove it from the press and adjust the usual screws before proceeding.

